# Current access and recruitment practices in nursing education institutions in KwaZulu-Natal: A case study of student nurses with disabilities

**DOI:** 10.4102/ajod.v8i0.429

**Published:** 2019-02-20

**Authors:** Selvarani Moodley, Gugu Mchunu

**Affiliations:** 1 School of Health Sciences, University of KwaZulu-Natal, South Africa; 2 School of Nursing and Public Health, University of KwaZulu-Natal, South Africa

## Abstract

**Background:**

While institutions of higher education may have increased access and accommodation for students with disabilities, institutions primarily providing nurse training in South Africa do not mirror the same practice.

**Objectives:**

Notwithstanding the integration of disability policies enacted in South Africa in 2010, a majority of people with disabilities are still excluded from the activities of society equally applicable to nursing education. This article describes the current access and recruitment practices for student nurses with disabilities (SNWDs) in nursing education institutions in KwaZulu-Natal to provide baseline data, which is largely absent in nursing institutions.

**Method:**

A concurrent mixed-method design using a multiple embedded case study approach was employed. This article presented phase 1 of the study, a quantitative survey of all private nursing education institutions (*n* = 27), complemented by individual, in-depth interviews with SNWDs (*n* = 10). Quantitative data were analysed using SPSS version 24, with a response rate of 78% (*n* = 21), whereas qualitative data were analysed using content analysis.

**Results:**

The findings revealed that the majority of private NEIs lack policy guidelines for recruiting SNWDs; however, other means of guidance is sought, for example, using the technical assistance. While NEIs were willing to recruit SNWDs, access to clinical sites, lectures, support systems and reasonable accommodation was challenging.

**Conclusion:**

Private NEIs are providing an inclusive education to all students including those with disabilities; however, they still have a long way to go in meeting the needs of SNWDs with regards to support and accommodation.

## Introduction

Higher education institutions have been encouraged to ensure access for and participation of people with disabilities; however, policies in the institutions of higher learning that train nurses still show gaps in meeting the needs of learners with disabilities. Previous education policies and practices have been focused along racial and political lines (Foundation of Tertiary Institutions of the Northern Metropolis [FOTIM] 2011), while students with disabilities were often not included in mainstream schools and special schools accommodated those who could afford it (Mutanga 2017). The post-apartheid era brought with it many changes, such as an equal opportunity for all learners, and although access for woman and black students to higher education has increased, limited consideration has been given to students with disabilities, resulting in them being excluded from higher education (Howell 2006).

Integration of disability in South Africa’s policies was enacted in 2010, with the aim of ensuring equal rights to all people in South Africa, including people with disabilities. There are various policies and frameworks in South Africa related to disability, which prevent discrimination, including: the constitution of South Africa (Republic of South Africa 1996); the *Employment Equity Act* (Republic of South Africa 1998); Disability Policy Guidelines (Republic of South Africa 2010) and the *Higher Education Act* 101 of 1997. However, specific policies related to higher education are very limited, fragmented and vague (FOTIM 2011; Gelbar et al. 2015). Despite these policies and regulations to protect and integrate people with disabilities, the majority of those with disabilities are still excluded from the activities of society, which applies to nursing as well (Republic of South Africa 2010).

Current policies reinforce an equitable education for all, including people with disabilities. The World Report on Disability (World Health Organization 2011) estimates that 15% of the world’s population have some form of disability, which can be equated to more than a billion people. According to the Disability Status report, 10.3% of working age people between the ages of 21 and 64 years in the United States have a disability (Erickson, Lee & Von Schrader 2012). In South Africa, 7.5% of the population is said to have disabilities (Statistics S A 2011). Less than 1% of the student population registered with the disability unit in South African higher education institutions, which equates to less than 2% of the overall student population (FOTIM 2011). These statistics suggest that students with disabilities are a minority group and, as such, can be subjected to discrimination and exclusion in a world created for the able-bodied. A review of disability literature in South Africa has indicated a limited number of studies in the field of disability, particularly in nursing education, which could indicate that it is still in the exploratory phase (Mutanga 2017). Studies on access usually refer to those in higher education (Fitchett 2015; Lyner-Cleophas et al. 2014; Matshedisho 2007). Furthermore, the FOTIM report indicates a low representation of students with disabilities in health sciences (FOTIM 2011). Various reasons could be presented for this under-representation and can all influence the recruitment of student nurses with disabilities (SNWDs) in nursing education institutions (NEIs), such as: negatives attitudes and stigma attached to disability (De Cesarei 2014); discrimination by nurse educators (Ryan 2011); recruitment policies; teaching and learning practices and the nursing curriculum.

Such discrimination overtly discourages students with disabilities from entering the nursing profession. Structures within the nursing profession itself, such as the absence of disability policy guidelines and in particular the clinical component of the undergraduate nurse training programmes, make it difficult for SNWDs to become registered or enrolled nurses. The clinical component is designed as a ‘one size fits all’ model for able-bodied students and is therefore looked at as being essential (Ryan 2011). Usually, the determination of appropriate accommodation in license-based programmes depends on whether the accommodation would pass the students’ development of ‘essential skills’ necessary for competent performance in the profession. Nursing programmes require student nurses to complete a minimum number of clinical practice hours per nursing programme before being entered for an examination. This places the NEIs, as well as educators, in a difficult situation, to ensure that the SNWDs meet the requirements and are deemed competent prior to completing the programme (Ashcroft et al. 2008).

An appraisal of available literature reveals limited studies on access and recruitment practices, or how well assessment and teaching practices cater for the needs of SNWDs in Higher Education Institutions (HEIs) and NEIs in South Africa (Matshedisho 2007). Even though universities have recently emphasised the role of the disability support services, increased access and support services are needed in universities (Ntombela & Soobrayen 2013).

This article reports on the results of a survey of all private NEIs and one public university in South Africa regarding their current access and recruitment practices. The study draws from Christensen and Rizvi’s (1996) socio-political model of disability, an updated ideology, which emphasises barriers in the education system that disable a person, and hence excludes them from the nursing profession. This view shifts the focus from the individual with the ‘deficit’, as explained in the medical model of disability, to examining how recruitment practices and teaching and curriculum practices pose a problem to SNWDs.

### Theoretical Framework: Framework for integrating student nurses with disabilities into nurse training programmes in KwaZulu-Natal nursing education institutions

The framework guiding the study was adapted from the Integrated Primary Health Care model by Sibiya and Gwele (2013) and Donabedian’s Systems Theory (Donabedian 1968). The phenomenon of interest in this study is integrating students with disabilities in nurse training programmes through various approaches, namely curriculum integration (Roxburgh et al. 2008), recruitment (Wood & Marshall 2010), access and accommodation (Roxburgh et al. 2008), clinical placement (Ashcroft & Lutfiyya 2013) and the academic environment (Tee et al. 2010). These five approaches are seen as the context and vehicle through which students with disabilities are incorporated into nurse training programmes. The concepts underlying the framework include an enabling environment, human resources, organisational support and productivity. Enabling environments are factors that contribute to the smooth functioning of an organisation such as human resources, organisational support services and collaboration (Sibiya & Gwele 2013). In the context of this study, an enabling environment is necessary to promote the smooth integration of SNWDs into nurse training and includes the access and recruitment practices at NEIs in the different nursing programmes, clinical placement and academic environment. Identifying barriers and enablers of integrating SNWDs in nurse training will promote an enabling environment.

### Problem statement

The number of NSWDs entering the nursing programme in South Africa is increasing, even though the exact number of these students is unknown (Neal-Boylan & Miller 2017). Despite the introduction of the Disability Policy Guidelines (Republic of South Africa 2010), students with disabilities are habitually effectively excluded from nurse training programmes, and hence from the nursing profession more broadly. This is mainly because of the absence of policy guidelines and the clear identification of the ‘essential skills’ needed to practise as a nurse. These can then be used to develop clear and realistic disability access guidelines for programmes, without violating the integrity of nursing preparation. This article, therefore, aims to describe the current access and recruitment practices for SNWDs in NEIs in KZN.

## Research methods and design

This article’s purpose was to describe the current access and recruitment practices for SNWDs in NEIs in KZN.

### Research question

What are the current access and recruitment practices for SNWDs in NEIs in KZN?

### Design

A descriptive, qualitative design using a concurrent mixed methods case study approach was employed. A quantitative design was employed to explore the current access and recruitment practices of SNWDs in NEIs. This article presents phase one of a much larger PhD study, a quantitative survey of all private NEIs (*n* = 27), complemented by a qualitative phase of individual in-depth interviews with SNWDs (*n* = 10). An instrumental descriptive, exploratory case study approach was used for this study (Yin 2014:10) because it enabled the researcher to look at disability in the context of nurse training to gain an in-depth understanding of the disability (Rule & John 2011:4).

### Research setting

This phase of the study took place in all private NEIs in KZN and one South African university. The private NEIs were purposively selected as they are more independent, and function as a stand-alone NEI, where decisions and procedures with regard to SNWDs can be made by the NEI itself without any outside influence.

### Sampling and participants

The list of NEIs from the South African Nursing Council (SANC) website was used as a sample frame (Polit & Beck 2014:180). A self-administered survey was administered to 27 NEIs (*n* = 27) and one public South African university included as a deviant sample, as these types of cases are difficult to obtain. The response rate of the survey was 78% (*n* = 21).

SNWDs were purposively selected (Polit & Beck 2014:179), because they met the inclusion criteria of having a disability, being a learner in one of the nursing programmes, and being willing to participate in the study.

### Data collection and analysis

#### Research instruments

Two types of data collection instruments were utilised in this study, namely survey questionnaires for all NEIs, strengthened by a semi-structured interview guide for SNWDs. The self-report, semi-structured survey questionnaire was adapted from a study by Wray, Gibson and Aspland (2007). The questionnaire consisted of two sections: Section A, to obtain demographic data, and Section B, to explore the current practices of integrating SNWDs into NEI training programmes in KZN, with regard to access and recruitment practices. Demographic information included gender, age, period of employment and highest level of education. Section B elicited information on the access and recruitment practices for SNWDs and consisted of both open-ended and closed-ended questions.

The semi-structured interview guide elicited information on the experiences of SNWDs with an emphasis on the access and recruitment practices at NEIs. The guide was flexible enough to allow the SNWDs to express themselves, and they were given an opportunity to raise concerns relevant to the study, even if it was not mentioned in the interview guide.

#### Individual interview

Individual face-to-face interviews were held with ten SNWDs at a date, venue and time convenient to them. All interviews were tape-recorded with their prior consent, and after explaining the ethical aspects including no monetary payment for participation.

#### Data analysis

The quantitative data collected were captured and subsequently analysed using the Statistical Package for Social Sciences (SPSS version 24). Descriptive statistics, such as frequencies and percentages, were used to summarise the data (Polit & Beck 2014:216). The data produced from the open-ended questions were analysed using thematic analysis as presented in this article.

Content analysis was used to analyse qualitative data obtained through the individual interviews with SNWDs (Hsieh & Shannon 2005). The individual interviews were transcribed verbatim; thereafter, manual coding was performed on the raw data. The data were divided into meaningful units for coding and categorising before they were themed.

### Ethical considerations

Ethical approval was obtained from University of KwaZulu-Natal (UKZN) Ethics Committee (reference number: HSS/1367/015D). Individual consent was obtained from each participant to participate in the study, and to have their voices recorded. Participants were reassured that their names would be kept confidential, and their right to self-determination, privacy, anonymity, confidentiality, fair treatment and protection from harm and discomfort were respected (Burns & Grove 2009; Emanuel et al. 2004).

## Results

The presentation of the findings was guided by the research questions and conceptual framework. Findings are presented according to socio-demographic information, current access and recruitment practices of nurse training programmes.

### Socio-demographic data

The majority of the principals of the NEIs were female 95.5% (*n* = 19), and only 9.5% (*n* = 21) were male. The ages of the principals of the NEIs ranged between 31 and 65 years. The majority of the principals (81%, *n* = 17) were over the age of 50 years, 9.5% (*n* = 2) were between the ages of 41 and 50 and 9.5% (*n* = 2) were between the ages of 31 and 40. The majority of the students with disabilities were female (*n* = 7) and the remaining three were male. SNWDs had a range of disabilities including vision impairment, hearing impairment, mobility impairment, dyslexia, physical disabilities such as missing digits and impaired hand and chronic conditions that were disabling such as arthritis.

### Current recruitment practices of student nurses with disabilities at nursing education institutions

This study findings revealed that the majority of the principals of private NEI (76.2%; *n* = 16) are directly involved in recruiting SNWDs. Eighty-one per cent (*n* = 17) of NEIs do not have internal policy guidelines for recruiting SNWDs; however, alternate sources are used as a point of reference, including the *Employment Equity Act* (Act No 55 of 1988); the Code of Good Practice (2015); Technical Assistance Guidelines (2015); the Skills Development Act (1998) and the Integrated National Disability Policy Guidelines (Republic of South Africa 2010).

Only 14.3% of students (*n* = 3) developed a disability while in the NEI, the remaining 85.7% were either not disabled or did not disclose their disability. The data collected from the NEIs contradicted data collected from the SNWDs. While one NEI indicated not applicable on the questionnaire, during the individual interviews with the SNWDs, the student revealed completing the enrolled nurse programme at the same NEI. The SNWDs further indicated that neither the lecturers nor the principal had noticed her disability, nor did she disclose it, as she feared she might be excluded from the nursing programme, as cited below:

‘All my lecturers didn’t notice I didn’t have a finger, even the principal, all of them they didn’t notice.’ (Participant 4, Martha, female, 24 years old)

A majority (85.7%, *n* = 18) of NEIs requested a declaration of health and/or disability prior to selecting students for the nursing programme; two (*n* = 2) NEIs requested it at the commencement of training, and at periodic intervals during the students’ training.

### Current practices of access to nursing education institution training programmes

Access to nurse training programmes is similar amongst the different NEIs, which requires all students to complete an application form, and attach their curriculum vitae. In some NEIs, selection is based on the results of a test students are obliged to take. This suggests that there is no discrimination between able-bodied and SNWDs and that all students are therefore treated equally.

One student noted:

‘Well, my experience on admission to the nursing college, […] I can’t really comment much about that. I didn’t have any problems, ’cause we follow a procedure like everyone else: you go and write a test, which consist[s] of [an] English essay and the Mathematics, and the Physical Science. So, we wrote that exam and then based on who got the highest mark[s], that’s how we got accepted’. (Participant 7, John, male, 37 years old)

In addition, the application form includes a section to be completed eliciting information about the students’ disability. This was not accurately completed, as SNWDs deliberately chose not to disclose their disability because of the fear of stigma and discrimination. For example, one hearing impaired student recounted:

‘I do feel that if I do put on the form that I have a disability, it may affect my chances of getting in, or lessen my chances I should say. Um, and because it doesn’t affect my work, I don’t find it necessary. Really, I’ll just tell them verbally that I have a hearing problem when I get there’. (Participant 2, Katy, female, 24 years old)

On the occasion where a decision needs to be made regarding a student’s fitness to train as a nurse, the NEI consults different key stakeholders. Table 1 indicates key stakeholders from the multidisciplinary team involved in deciding on the student nurse’s fitness to undertake a nursing programme: nursing service manager (12.5%, *n* = 2); principal of the college (82.4%, *n* = 14); college council (12.5%, *n* = 2); doctor (52.6%, *n* = 10); human resource practitioner (12.5%, *n* = 2) and the applicant themselves (18.3%, *n* = 3). SNWDs are sometimes referred to the occupational health nurse (30.8%, *n* = 4), social worker (8.3%, *n* = 1) or psychologist (33.3%, *n* = 4).

**TABLE 1:** Key stakeholders involved in decision-making regarding the students’ fitness to undertake nurse training.

Key stakeholders	Yes	%	No	%	*n*
Nursing service manager	2	12.5	14	87.5	16
Principal of the college	14	82.4	3	1.6	17
College council/senate/campus board	2	12.5	14	87.5	16
Doctor/medical advisor	10	52.6	9	47.4	19
Human resource practitioner	2	12.5	14	87.5	16
The applicant	3	18.3	13	81.3	16
Occupational health nurse	4	30.8	9	42.9	13
Social worker	1	8.3	11	91.7	12
Psychologists	4	33.3	8	66.7	12

**TABLE 2:** Access to clinical sites.

Type of reasonable accommodation	Yes	%	No	%	*n*
Providing modified equipment	2	13.3	13	86.7	15
Modify workstation, for example OSCE	2	14.3	12	85.7	14
Make existing facilities accessible to SNWDs	5	35.7	9	64.3	14
Modify test times	3	21.4	11	78.6	14
Allow for special leave on duty	3	21.4	11	78.6	14
Provide additional support	8	57.1	6	42.9	14
Provide training or retraining	4	28.6	10	71.4	14
Provide counselling	1	7.7	12	92.3	13

OSCE, objective structured clinical examination; SNWDs, student nurses with disabilities.

A common thread that emerged amongst SNWDs was communication barriers, especially in hearing impaired SNWDs when lecturers spoke in a very soft tone, as indicated in the following:

‘…especially at college, when writing the notes while the teacher is busy educating us. Sometimes I can’t hear. She can’t write the notes, she so difficult’. (Participant 8, Isobel, female, 41 years old)

Another student noted:

‘They understood, and they allowed me to sit in the front. They spoke louder and usually they would give me eye contact while lecturing, and that helped a lot. There were some strict ones that don’t want to hear anything you have to say. Hey, they just want to lecture and leave, so I didn’t want to bother them with my situation’. (Participant 2, Katy, female, 24 years old)

The above quote suggests that while some educators are willing to assist and accommodate SNWDs, other educators are not so accommodating.

Nursing education institutions perceive students with chronic conditions, such as epilepsy, to have a disability and reported that these students needed more time to grasp the teaching material as compared to other able-bodied students. The NEIs further reported that students on chronic medication, such as anti-epileptics, experienced side effects for example altered thought processes, decreasing a student’s concentration span.

### Access to support systems

A percentage of 52.4% (*n* = 11) of the NEIs indicated having lecturers with specialist skills and training to support and manage SNWDs, the remaining 47.6% (*n* = 10) indicated a lack thereof. Specialist skills were not specified, but most NEIs indicated that educators were registered nurses, and therefore perceived them to be able to manage SNWDs. A strong source of support was other colleagues, and friends of SNWDs, who were more than willing to assist by sharing notes, for example.

### Access to reasonable accommodation

More than half of the NEIs surveyed did not provide support in the form of modified equipment (86.7%, *n* = 13), modified workstations during an objective structured clinical examination (85.7%, *n* = 12), made existing facilities accessible to SNWDs (64.3%, *n* = 9), modified test times (78.6%, *n* = 11), allowed for special leave on duty (78.6%, *n* = 14), provided training or retraining (71.4%, *n* = 10) and provided counselling (92.3%, *n* = 13).

Accessing the clinical area for SNWDs was challenging because of a lack of funds for transport, as SNWDs already had to pay fees for training at NEIs. Sometimes the only source of income for SNWDs is a state grant.

### Physical infrastructure access

A majority of NEIs are accessible to SNWDs, but with some limitations, such as a lack of escalators, as quoted below:

‘I would just love the schools to accommodate people with disabilities; I would say maybe lifts, because at first, I didn’t know how to walk up and down the stairs. In the first week, I used crutches, but as I’m saying […] it was difficult, it was difficult for me. But at the same time, I was […] the tutors were waiting for me at the top, I need to go up … I need to have my bag, … Because she’ll [educator] be […] waiting for me at the top’. (Participant 3, Rachael, female, 23 years old)

In NEIs where escalators were present, access to certain lectures not on the ground floor was denied, for example lectures held in the skills laboratory. Even though lecturers were willing to change the venue to accommodate SNWDs, they were unable to move the skills laboratory because of availability of specialised equipment, resulting in SNWDs missing those lectures, as well as clinical time in the skills laboratory. This is a revealing and critical finding, as it demonstrates the disjointed nature of disability support in programmes that go outside of the regular classroom lecture setting. This finding is key and is in violation of the equity of access practices.

### Barriers to accessing nursing programmes

A small minority of private NEIs (23.8%, *n* = 5) revealed a lack of funds to be a barrier for accommodating and training SNWDs, especially with regard to purchasing modified equipment. The lack of funds is often given as a reason for not providing accommodation but, in reality, it is an unacceptable excuse, since the federal guiding documents such as the Disability Policy Guidelines (2010) require equity of access, and never said ‘if affordable for the institution’ (Constitution of SA 1996). Physical environmental barriers, such as inaccessible buildings, social barriers such as stigma, stereotyping and attitudes were also a barrier in a small minority of NEIs (19%, *n* = 4). Other barriers included lack of knowledge in reasonable accommodation found in only 9.5% (*n* = 2) of NEIs; lack of collaboration between college staff and hospital staff (19%, *n* = 4) and lack of skills/availability of educators to manage SNWDs (19%, *n* = 4). In addition, students themselves reported the course as being stressful, and sought assistance and counselling from lecturers.

## Discussion and findings

### Socio-demographic findings

The majority of the principals were female, as seen in studies by Christensen (2017), which found nursing to be a female-dominated profession. The ages of principals of the NEIs ranged between 31 and 65 years, with a majority of the principals being above the age of 50 years (81%, *n* = 17). This could be consistent with nursing seen an aging population, or it could also mean that posts such as these are reserved for the more experienced and skilled professionals (Phillips & Miltner 2015; Vance 2011). The global increase in chronic conditions associated with disabilities coupled with an aging nursing workforce increases the risk for disabilities amongst nurses as well (World Health Organization 2011).

### Current recruitment practices of student nurses with disabilities at nursing education institutions

The common thread that emerged from all participants was that gaining access to the nurse programmes was not difficult. The different NEIs have a common procedure used to recruit all students, including SNWDs, which includes completing an application form and attaching a curriculum vitae. The study’s findings suggest that the majority of NEIs lacked internal policy guidelines for recruiting and integrating SNWDs; however, alternate sources were used as a point of reference. While the majority of the principals of the NEIs are directly involved in recruiting SNWDs, when decisions need to be made regarding a student’s fitness to undertake the nursing programme, different key stakeholders from the multidisciplinary team are consulted.

The findings of the survey indicate that a large majority of students were either not disabled or did not disclose their disability. It is interesting to note that while one NEI indicated ‘not applicable’ on the survey questionnaire, during the individual interview with the SNWDs, this participant revealed having completed the enrolled nurse programme with the NEI, suggesting a disjuncture between the findings. This study further suggests that SNWDs were afraid to disclose their disability, for fear of discrimination, and hence being excluded from the course, which concurs with the findings of a study by Ryan (2011), which found that acquiring a place in the Bachelor of Nursing programme is one of the major hurdles for students with disabilities.

It is important to note that even though the NEIs requested a declaration of health and/or disability at periodic intervals during the students, training, none of the NEIs requested a physical examination during the recruitment process. The majority of the students had hidden disabilities, which made it easier for the SNWDs to hide their disabilities, until they were accepted into the nurse training programme, and only disclosed their disability once they secured a place in the programme. In some instances, the lecturers only became aware of the disability when meeting the students, usually in class or during clinical accompaniment, which resonates with findings from Aaberg (2010). Other students went through their entire course of training without ever disclosing their disability.

This study’s findings concur with previous findings of Mosia and Phasha (2017) that SNWDs lack support services and assistance is largely absent in the majority of the private NEIs. Other findings suggest that there is a lack of knowledge and experience to support SNWDs (Aaberg 2010). A huge contributory factor to this could be the absence of policy guidelines to guide the access and integration of SNWDs in NEIs, which results in the provision of support being situational rather than systemic (Mosia & Phasha 2017). It is important for nursing departments to develop support systems in collaboration with disability units, disability support organisations and professional nursing organisations, which can help identify what skills are essential as is, and what skills can be accommodated or adjusted to address access needs. Relying solely on the disability unit support, usually designed for classroom-based learning, may not suffice (Coriale, Larson & Robertson 2012). Previous research findings exploring the experiences of registered nurses while they were in nursing programmes revealed that accommodations approved by the disability unit were sometimes not carried out by the nursing department, as in the case of the student with a urinary problem who was not allowed to go to the toilet every 3 hours as required (Neal-Boylan & Miller 2017).

The NEIs indicated a lack of funds to purchase specialised equipment or to recruit additional staff members, which confirmed the findings of a study by Emong and Eron (2016) that private hearing impaired students lacked funds for enlisting the help of an interpreter and usually shared the interpreter of a student funded by the government. In addition, students with disabilities in this study lacked funds for travelling to clinical sites, as a state grant was sometimes the only source of income.

On a more positive note, SNWDs depend heavily on other colleagues for assistance, such as note taking (Mosia & Phasha 2017). Similar findings by Neal-Boylan and Miller (2017) describe how colleagues were committed to assisting one another, as students felt they ‘were in this together’. SNWDs also depend on their family members for support and encouragement, which motivates them to persevere in the nursing programmes.

### Current practices of access to nursing education institutions training programmes

The findings of this study evidence adequate access to the physical environment of NEIs, such as well-designed buildings with ramps and escalators; however, the escalator was often not in good working order. This prevented SNWDs from accessing lectures such as those in the skills laboratory located above the ground floor. Even though lecturers were willing to relocate the venue for normal classroom lectures, specialised rooms such as the skills laboratory could not be moved, because of specialised equipment and resources.

The lecture method is the most common teaching strategy used, which requires face-to-face contact. This was a challenge, as the lecturer sometimes walked around the class, which made it difficult for hearing impaired students to lip read. Hearing impaired students, in addition, use facial expressions and facial cues when interpreting messages, and this proved difficult when educators spoke and wrote on the board at the same time, which coincides with the findings of Emong and Eron (2016). In addition, lecturers sometimes do not write notes on the board, nor do they have a PowerPoint presentation, which made it difficult for SNWDs to follow the lecture. Further to this, visually impaired students were sometimes unable to read notes on the board and have to rely on other means of assistance, for example getting their colleagues and friends to help (Mosia & Phasha 2017). Some lecturers provide notes, but these were also difficult to read, and can be equated to the lack of resources, such as large print books, cited by Mosia and Phasha (2017) in their study, which examined students with disabilities’ access to higher education institutions in Lesotho.

### Reasonable accommodation

Reasonable accommodation provided to SNWDs includes modifying equipment, workstations, test times, providing additional support, training or retraining and counselling. Modified test times varied at different NEIs, between 15 and 30 min extra per test and/or examination, because of the absence of policy guidelines to guide NEIs when recruiting SNWDs. Emong and Eron (2016), in a study in Uganda exploring disability inclusion in higher education, reveal similar findings, namely the consequence of a lack of policy to support students with disabilities, meaning support for students with disabilities was situational rather than systemic. The university as a body of higher education has made considerable efforts to include and accommodate SNWDs, as compared to the other private NEIs, mostly nursing colleges. This could be because of the fact that the university also has a disability unit attached to readily assist with special needs of students with disabilities.

### Barriers to accessing nursing programmes

The financial implications of recruiting SNWDs are a challenge for NEIs, which do not have adequate funds to purchase any new and specialised equipment/resources, such as employing a sign language interpreter for hearing impaired students. Hearing impaired students also experienced challenges when lecturers spoke in a soft tone or when lecturers walked around the class while lecturing. Hearing impaired students expressed concern that they needed to see the lecturer to be able to lip read. They added that facial expression also played a role during communication. The researcher proposed these aspects be included in the guidelines for nurse educators to have a source of reference to guide their practice. The only source of income for students with mobility impairment is a minimal state grant, which makes it difficult for them to travel to the clinical site daily using public transport to ensure their clinical hours are met. Mobility impaired students also experienced difficulty accessing lecture venues. Barriers to the lecturers for vision impaired students were the lack of notes and/or PowerPoint presentations from some lecturers.

The rational explanation for the lack of support and provision of reasonable accommodations is the lack of disclosure by students themselves. The lack of guidelines further compounds these challenges as nurse educators lack a reference document to ensure support and reasonable accommodation is provided to all students in a fair and consistent manner preventing any form of discrimination. Hence, the researcher proposes including a standardised procedure for recruitment and selection of all learners including SNWDs, which should encourage voluntary disclosure within an enabling and supportive environment by a multidisciplinary disability committee.

In addition, students themselves reported the course as being stressful, and sought assistance and counselling from lecturers. The author concludes that determining the physical skills essential for the nursing profession and identifying skills that are able to apply accommodation without harming the integrity of professional competence may help reduce barriers.

## Limitations of the study

These study findings indicate that SNWDs still choose to hide their disability because of disclosure. The number of students identified in this study is limited to those students who disclosed their disability, and hence the study cannot represent the total population of SNWDs in private NEIs. In addition, this study was conducted in private NEIs and one public university only, and for that reason, it should be interpreted with caution and not be generalised to all NEIs in South Africa.

## Conclusion and recommendations

Recruitment practices, teaching and learning practices, and the nursing curricula can all influence the recruitment of SNWDs in NEIs (FOTIM 2011). The recruitment practices for all students were the same, with no specific process for SNWDs suggesting no discrimination in recruiting of SNWDs. When a decision was needed to be made regarding a student’s fitness to practise, members of the multidisciplinary team were contacted, but principals of the NEI were largely responsible, suggesting a lack of coordination between the multidisciplinary disability support team and the NEI principals as a key finding. Recruiting SNWDs was easy, but access to lecture rooms, curriculum, support, reasonable accommodation and clinical sites was limited.

While every effort was made to include SNWDs in NEI programmes, accommodating SNWDs was situational rather than systemic, and in the absence of clear policy guidelines, some lecturers were more than supportive, while others were not. SNWDs preferred to hide their disabilities until they secured a space in the programme, and only disclosed their disability when reasonable accommodation was required, while some students went through the entire programme without informing the NEI about their disability. It is significant to note that countries like the United States of America have federal laws such as the Americans with Disabilities Act 1990, amended in 2008, and Section 504 of the Rehabilitation Act of 1973 that protect people in higher education from any form of discrimination and require higher education accommodation as a civil right (Americans Disability Act 1990). The author of this article, therefore, suggests that the absence of policy guidelines for integrating SNWDs in nursing programmes is a key contributory factor for not meeting the 2% target of employing people with disabilities in South Africa.

The researcher makes the following recommendations: further collaborative planning of the NEI principals and disability support staff is needed to develop the mutually agreed upon support system to use. Barriers in nurse training environments need to be identified and the ‘essential functions’ of nursing programmes need to be decided on by key stakeholders involved in nursing education. Continuing professional development of educators on the integration of SNWDs and inclusive teaching practices in the classroom, as well as reasonable accommodation in the practice-based setting (Lombardi, Murray & Dallas 2013) are some of the recommendations. The development of policy guidelines to ensure consistency and prevent unfair discrimination on SNWDs ought to be undertaken (Mutanga 2017). Marks and McCulloh (2016) reveal that best practices for nursing in the 21st century should include accommodations to improve clinical experience using technology to transform nursing education, research and practice.
